# Antidepressant Use Before, During, and After Pregnancy

**DOI:** 10.1001/jamanetworkopen.2024.57324

**Published:** 2025-01-30

**Authors:** Claire Boone, Carla Colina, Devin Pope

**Affiliations:** 1 Department of Economics, McGill University, Montreal, Quebec, Canada; 2 Department of Equity, Ethics, and Policy, McGill University, Montreal, Quebec, Canada; 3 Stanford Graduate School of Business, Stanford, California; 4 University of Chicago Booth School of Business, Chicago, Illinois

## Abstract

This cohort study investigates rates of antidepressant use among women 24 months before and after the birth of a child.

## Introduction

Women’s lifetime risk of depression is highest during and after pregnancy, and those with a history of depression have particularly high risk of relapses during this period.^1^ Choosing whether to continue taking antidepressants during pregnancy can be complicated. Although evidence is limited, there are safety concerns regarding in utero exposure to some antidepressant medications.^1^ However, untreated maternal depression can have severe long-term consequences for mother and child.^2,3,4^

For women who choose to discontinue antidepressant medication during pregnancy, current guidelines recommend substituting with psychotherapy.^5^ Clinicians therefore play an important role in helping women make informed decisions about mental health care during pregnancy. We provide descriptive evidence of the choices women and their clinicians make about depression treatment during pregnancy.

## Methods

This cohort study follows the STROBE reporting guideline and was deemed not human participants research by the University of Chicago Institutional Review Board. Analyses were conducted January to November 2024.

We used Merative MarketScan Research Databases, which contain claims data for a large sample of privately insured individuals in the US. The study included women who gave birth between 2011 and 2017 and had prescription drug insurance coverage the month they gave birth and 24 months before and after (eMethods in Supplement 1). We also analyzed women’s spouses if the spouse was on the same insurance (56.5% of women) (eMethods in Supplement 1).

We measured antidepressant medication fills and psychotherapy claims of women and their spouses for 2 years before and 2 years after the birth of a child (2009-2019). For each outcome, we plotted the unadjusted monthly share of individuals with claims relative to the birth month separately for women and spouses (eMethods in Supplement 1).

Our data differ from previous studies, which have primarily measured antidepressant use via self-reported surveys or have examined relatively small samples.^4^ We further add to the literature by measuring psychotherapy claims to provide a more complete description of depression treatment. Analyses were conducted using Stata statistical software version 17 (StataCorp).

## Results

Among 385 731 included women (mean [SD] age at childbirth, 31.8 [5.4] years), 74.8% were employed and the mean (SD) income was $84 577 ($39 676). We found that 4.3% of women filled an antidepressant prescription in the year before pregnancy and 2.2% filled an antidepressant prescription during pregnancy, a reduction of 48.8% (Figure, A). We find no similar change in antidepressant use among 217 877 spouses, suggesting that this behavior was not associated with other, co-occurring changes in the couple’s life (Figure, A). Women did not appear to substitute with psychotherapy; during pregnancy, we found a slight decrease in psychotherapy claims relative to a trend line (Figure, B).

**Figure.**
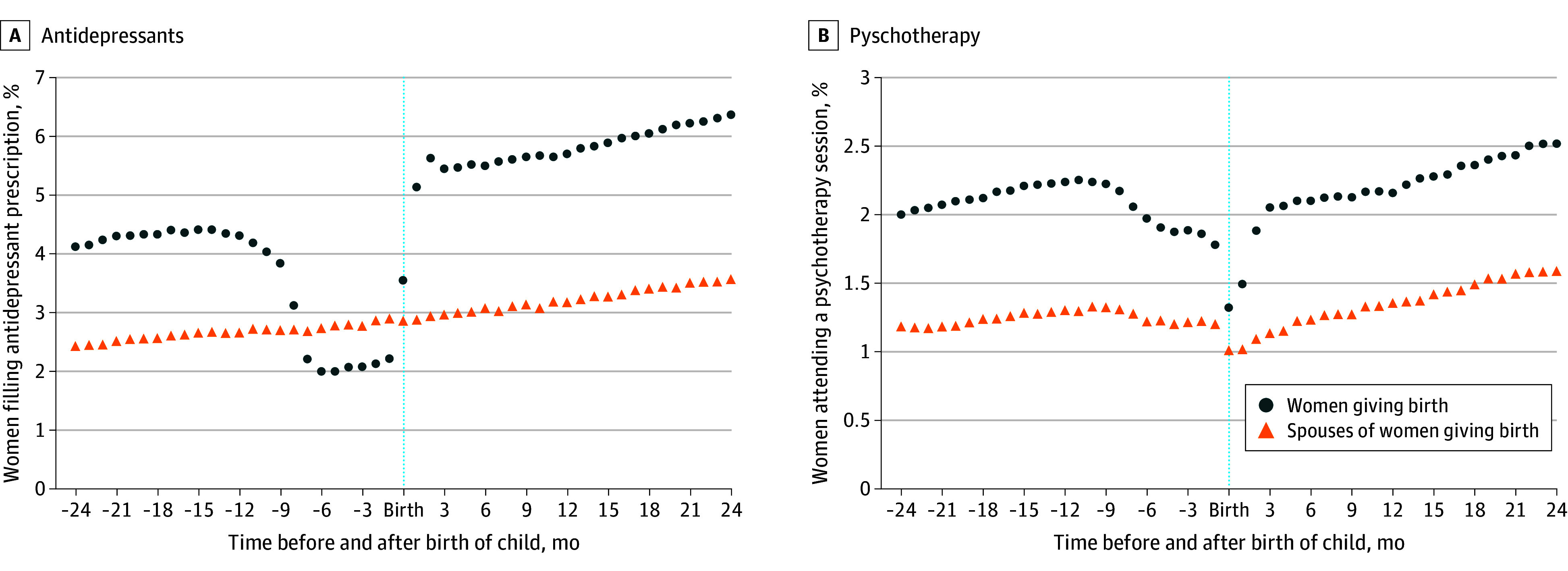
Changes in Claims for Depression Treatment Changes are shown among women giving birth and their spouses 24 months before and 24 months after the birth of a child, including antidepressant refill rates for all brands combined (A) and claims for psychotherapy (B).

Within 1 month of giving birth, women’s mean medication fills returned to the prepregnancy trend line, suggesting that women valued treatment for depression but were choosing to avoid antidepressant use during pregnancy. However, given the time delay for antidepressant medication to function, restarting medication after pregnancy may leave many women effectively untreated during the high-risk postnatal period.

## Discussion

In this cohort study, we documented a large decrease in antidepressant use without an accompanying increase in psychotherapy during pregnancy. These findings, coupled with evidence of mental health challenges during and after pregnancy,^1^ suggest the need for increased focus on and discussion about mental health treatments by pregnant women and their clinicians.

A limitation is that we did not observe medication adherence and instead relied on refills in claims data. While imperfect, claims are less likely to overestimate medication fills than self-report data and retrospective surveys.^6^ Another limitation is that the data do not include survey evidence to better explore the reasons why some women discontinued their medication. Additionally, our data include only privately insured individuals in the US, and so findings may not generalize to other populations.

## Notes

Supplement 1.
eMethods.

Supplement 2.
Data Sharing Statement
