# Supplementary material for: Antidepressant Use Before, During, and After Pregnancy
Source: JAMA Netw Open. 2025 Jan 30;8(1):e2457324. doi: 10.1001/jamanetworkopen.2024.57324 (PMC11783193; doi:10.1001/jamanetworkopen.2024.57324)
Supplement: Supplement 1. — eMethods. [file jamanetwopen-e2457324-s001.pdf]

Supplemental Online Content

Boone C, Colina C, Pope D. Antidepressant use before, during, and after pregnancy. *JAMA Netw Open*. 2025;8(1):e2457324. doi:10.1001/jamanetworkopen.2024.57324

eMethods.

This supplemental material has been provided by the authors to give readers additional information about their work.

© 2025 Boone C et al. *JAMA Network Open*.

eMethods.

Data & Sample

We used data from Merative MarketScan Research Databases, an administrative claims database that includes inpatient and outpatient claims, outpatient prescription claims, clinical utilization records, and healthcare expenditures. These data represent the medical experience of insured employees and their dependents who are covered under their insurance. The sample comprises active employees, early retirees, COBRA continues, and Medicare-eligible retirees with employer-provided insurance.

Specifically, we link data at the individual level across the outpatient dataset which contains psychotherapy claims (2009-2019), the inpatient dataset which contains births (2011-2017), and the prescriptions dataset which contains antidepressant prescriptions and fills (2009-2019). While births data are also available through 2019, our analysis requires that two years of claims data are available after the birth of the child, so we include births only to 2017.

We use the inpatient claims dataset to identify 388,182 adults (age≥18 years) who had prescription drug insurance coverage for the month they gave birth, and the 24 months before and after, and whose diagnosis was one of the following:

- Cesarian Section with complication or comorbidity (CC) or a major complication or comorbidity (MCC)
- Cesarian Section without CC/MCC
- Vaginal Delivery with Sterilization and/or Dilation and curettage (D&C)
- Vaginal Delivery with Major operating room (OR) procedures except for Sterilization and/or D&C
- Vaginal Delivery with Complicating Diagnoses
- Vaginal Delivery without Complicating Diagnoses

© 2025 Boone C et al. *JAMA Network Open*.

We exclude 594 women who appear more than once on the same admission date or who were admitted with female companions (mostly their mothers) because we cannot discern who is giving birth. However, women who gave birth and were admitted with a female spouse are included in our analysis.

Last, we exclude 1,857 males who gave birth during our study period. While our results are qualitatively unchanged if we include these individuals in our main figures, we exclude them because statistics on depression in trans men are very different than those of depression in women; a nuance we did not have space to elaborate on in this short report.

Our final sample is 385,731 women and 435,148 births.

We use the family ID of the women giving birth to identify their spouses. We restrict the spouse sample to individuals 18 or older whose insurance plan captures drug claims and had complete insurance coverage during the study window. We identify 217,902 spouses with these characteristics. Women who meet our inclusion criteria were included whether they had a spouse or not.

We include spouses to help rule out the possibility that other temporal trends might be influencing individuals' decisions regarding antidepressant and psychotherapy use. For instance, if women altered their use of antidepressants or psychotherapy due to events happening at the same time as their pregnancy, we might expect to see similar trends in their spouses. By plotting spouses' depression treatment patterns before, during, and after pregnancy, we aim to strengthen the descriptive analysis and provide a clearer understanding of whether these changes are specific to pregnancy.

Table S1 describes women who gave birth and their spouses included in our analysis.

© 2025 Boone C et al. *JAMA Network Open*.

Demographic variables

As our analysis is based on an administrative insurance claims dataset, we have limited access to demographic variables for women and their spouses. In the claims data, we observe age, employment sector, and Metropolitan Statistical Area (MSA) of residence, but not race or ethnicity. We report average age, employment status, and average income for the year a woman gave birth. Employment status was constructed using individuals' employment sector. If an employment sector was reported we assign employed =1, 0 otherwise. Therefore, individuals may not have employment data if they are not employed, if they are a student, or if they did not report their employment sector, but we cannot differentiate between these situations.

To recover average income, we linked Bureau of Labor Statistics' (BLS) Quarterly Census of Employment and Wages data to our administrative claims data. For each individual, we linked BLS annual wage for a MSA, employment sector, and year. Individuals who did not have employment sector data are excluded from the average wage calculations (25.2% of women, and 20.8% of spouses). Conditional on an individual having employment information and being identified as living in an MSA, the BLS wage data was available at the MSA-sector-year level for 62% of the sample (regardless of whether they gave birth or are a spouse). The remaining 38% were assigned wage data at the state-sector-year level.

Outcome measures

Our outcome measures are defined as a binary variable for whether an individual has filled an antidepressant prescription in a given month or whether an individual has incurred a psychotherapy service in a given month. We define this outcome among individuals who ever filled an antidepressant or had a psychotherapy claim in the 24 months before they or their spouse gave birth.

© 2025 Boone C et al. *JAMA Network Open*.
