# Supplementary material for: Antidepressant Use Before, During, and After Pregnancy
Source: JAMA Netw Open. 2025 Jan 30;8(1):e2457324. doi: 10.1001/jamanetworkopen.2024.57324 (PMC11783193; doi:10.1001/jamanetworkopen.2024.57324)
Supplement: Supplement 2. — Data Sharing Statement [file jamanetwopen-e2457324-s002.pdf]

Data Sharing Statement

Boone. Antidepressant Use Before, During, and After Pregnancy. *JAMA Netw Open*.
Published online January 30, 2025. doi:10.1001/jamanetworkopen.2024.57324

Data
Data available: No

Additional Information
Explanation for why data not available: These data are proprietary and we are not able to make them available.
